# The Impact of Maternal Self-Efficacy and Oral Health Beliefs on Early Childhood Caries in Latino Children

**DOI:** 10.3389/fpubh.2017.00228

**Published:** 2017-08-28

**Authors:** Anne R. Wilson, Matthew J. Mulvahill, Tamanna Tiwari

**Affiliations:** ^1^School of Dental Medicine, University of Colorado Anschutz Medical Campus, Aurora, CO, United States; ^2^School of Medicine, University of Colorado Anschutz Medical Campus, Aurora, CO, United States

**Keywords:** self-efficacy, health beliefs, maternal behavior, Hispanic Americans, caregivers, dental caries, child

## Abstract

**Objectives:**

Latino children experience one of the highest rates of early childhood caries requiring interventions based on valid conceptual frameworks. The Health Belief Model has relevance as a predictor of compliance with health recommendations based on perceptions of a health condition and behaviors to avoid the condition. The model encompasses four perceptual constructs (susceptibility, severity, benefits, barriers) and, for complex conditions, includes self-efficacy as an extended model. This study evaluated individual (self-efficacy and health beliefs) and cultural (acculturation status) level factors and the inter-relationship to determine if items assessed for the Extended Health Belief Model (EHBM) were valid measures of maternal factors.

**Methods:**

A cross-sectional study was conducted with 100 mother–child dyads at the Dental Center of Children’s Hospital Colorado, Aurora, CO, USA. Participating mothers completed a survey in English or Spanish with items from the Basic Research Factors Questionnaire encompassing sociodemographic characteristics, oral health knowledge and behavior, and psychosocial measures including the EHBM. Language preference was a proxy for maternal acculturation. Children were examined to measure decayed, missing, and filled tooth surfaces. Internal consistency reliability of each subscale was evaluated using Cronbach’s alpha. Convergent validity was assessed using linear regression to evaluate the association of the EHBM subscales with oral health-related measures and language preference.

**Results:**

The benefits and self-efficacy scales reflected good reliability. Maternal education was the strongest predictor of health beliefs with significant associations for barriers, benefits, and susceptibility. Perceived benefits increased with each additional year in the household. There was a significant association between maternal oral health knowledge and higher perceived benefits and increased self-efficacy, and the same was found for higher knowledge of dental utilization which was also associated with children perceived as having increased susceptibility to early childhood caries. Less acculturated participants perceived more barriers to behavioral adherence and fewer barriers as knowledge increased. As dental utilization knowledge improved for Spanish-speaking participants, they perceived greater benefits from adherent oral health behavior compared to English-speaking participants.

**Conclusion:**

Items assessed for the EHBM were valid as measures of maternal factors influencing children’s oral health outcomes in a Latino population.

## Introduction

Among disadvantaged children, oral health disparities have persisted with racial and ethnic groups disproportionately affected by early childhood caries. Epidemiologic data from national surveillance studies indicate Latino children experience the highest prevalence of early childhood caries compared to other racial/ethnic groups (1–3). Models developed to improve oral health outcomes among young children have traditionally focused on biological influences with poor predictive results as up to 85% of health outcomes are associated with social determinants influencing an individual’s response to adverse health conditions (4, 5). Accordingly, emphasis has extended to encompass social determinants at the caregiver level as integral influences on oral health outcomes in children including knowledge, health beliefs and attitudes, stress, self-efficacy, social network strength, and acculturation status (6, 7). Addressing the impact of social determinants among disadvantaged children requires effective interventions based on valid conceptual frameworks. Development of a validated caregiver instrument assessing a range of constructs related to children’s oral health outcomes has value for the Latino population and others burdened by social inequalities in oral health. This study aimed to evaluate both individual (self-efficacy and health beliefs) and cultural (acculturation status) level factors and the potential inter-relationship between these factors/levels in mothers of Latino children.

The influence of acculturation on disparities in systemic health has been well established, yet the impact on oral health has been less studied (8). Acculturation is measured in various ways including scales (9, 10) or proxy measures that may include length of stay in the host locale, nativity, generational status, and language preference or competency (8, 11, 12). The process of acculturation involves behavioral change and integration of new beliefs with those from the original culture (13). Latinos with high acculturation status are more likely to receive health care compared to those with low acculturation (14). High acculturation status in Latino caregivers has been associated with increased dental utilization for children (15). Studies suggest that Latino children in non-English primary language households experience dental disparities with poor oral health and unmet dental needs as well as a higher need for interpreters due to language barriers (16). Low acculturation of caregivers and proximate factors including decreased education may contribute to reduced access to oral health care in Latino children (17). Latinos with low acculturation also face barriers related to decreased knowledge of insurance programs and services, cultural differences in time orientation and unfamiliarity with expectations related to scheduling health visits, and unease in accessing health care due to concerns about citizenship (18). Despite social disadvantage in U.S. immigrant children, research has minimally examined the effects of caregivers’ acculturation on oral health outcomes and the relationship with social determinants (19).

Within health promotion research, the Health Belief Model (HBM) is one of the earliest and most widely used conceptual models (20, 21). The HBM posits that health behavior is determined by an individual’s perceptions of a health condition and actions to avoid the condition. The model (20) includes four key constructs: perceived susceptibility, perceived severity, perceived benefits, and perceived barriers. The HBM purports that for individuals to follow health recommendations, they must perceive: they are susceptible to developing a given disease (higher perceived susceptibility), the disease is serious (higher perceived severity), there are more benefits to engaging in adherent behavior (higher perceived benefits), and fewer potential impediments to engaging in positive health behavior (lower perceived barriers). These perceptions are potentially influenced by individual factors including demographics, knowledge, behavior, and cultural factors.

Earlier applications of the HBM were used to predict simple health behaviors, such as one-time immunizations. Eventually, the model was applied to complex health concerns requiring long-term behavioral modification. In 1988, an extended model was introduced, which combined the concept of self-efficacy with the HBM constructs. As a behavioral determinant, self-efficacy reflects the extent to which a person feels capable of successfully engaging in recommended health behaviors (22). Although the concept of self-efficacy stems from the Social Cognitive Theory (22), it was integrated (23) with the HBM because of reliability as a predictor of health behavior (24–26) and a theoretical connection to the HBM construct of perceived barriers (20, 23). The Extended Health Belief Model (EHBM) has been widely applied to a range of medical concerns in health promotion research (27–29), yet application in oral health research has been limited. The EHBM has relevance for early childhood caries, a chronic and behaviorally mediated disease requiring engagement in complex behaviors. Research has not addressed oral health disparities among Latino children relative to the EHBM and maternal influences (30). Hence, this study assessed the internal consistency and reliability of the EHBM measures in relation to individual (knowledge, behavior, and oral health outcomes) and cultural level factors (acculturation) and their importance and potential inter-relationship.

## Materials and Methods

### Study Design

A cross-sectional survey was conducted with 100 Latino mother–child dyads. All recruited children were patients at the Dental Center of Children’s Hospital Colorado in Aurora, CO, USA, and accompanied by their mothers. The protocol was approved by the Colorado Multiple Institutional Review Board. Participants provided written informed consent and Health Insurance Portability and Accountability Act authorization prior to study participation. The study protocol was described in an earlier report (31) and only key features are presented.

### Participants

Enrollment criteria required participating mothers to be at least 18 years of age and the primary caregiver for a child under 6 years of age based on the case definition of early childhood caries as birth up to 72 months of age (32). Participating mothers were given the option to sign the consent form and complete the paper-based survey in English or Spanish and certified translators provided study information to participants with a stated preference for Spanish. All participating children in the study received an oral examination to measure decayed, missing, filled, and surfaces in the primary dentition (dmfs). Study methods were consistent with the STROBE guidelines for cross-sectional studies.

### Procedures

Participating mothers completed a questionnaire adapted from the Basic Research Factors Questionnaire (BRFQ). Development of the BRFQ survey (33) was a collaborative effort involving three Oral Health Disparities Centers funded by the National Institute of Dental and Craniofacial Research to address the excessive burden of oral disease in racial/ethnic minority populations and other disadvantaged communities. The BRFQ survey items encompassed demographics as well as caregivers’ oral health knowledge and behavior, and other psychosocial measures jointly specified or developed by investigators from the Oral Health Disparities Centers: University of Colorado Denver, Boston University, and University of California San Francisco. The BRFQ is available in English and Spanish and has been administered to diverse populations by the three Oral Health Disparities centers.

Survey items related to knowledge and behavior were developed to address twelve specific content areas. These areas were identified by the cross-center Behavioral Intervention Workgroup, which was charged with specifying the counseling messages that should be incorporated into each center’s clinical trials targeting disparities in early childhood caries. Key messages that were identified to guide development of survey items related to knowledge and behavior addressed the following content: (1) cavities are caused by germs, (2) baby teeth are important, (3) brush teeth every day, (4) use fluoride toothpaste, (5) help children brush up to age six, (6) limit sweet foods and drinks, (7) take your child to the dentist, (8) take care of your own teeth, (9) no bottles or sippy cups in bed, (10) do not share germs, (11) wean your child from the bottle by 1 year, and (12) look at your child’s teeth once a month for spots or problems.

#### Oral Examination

A calibrated pediatric dentist conducted visual screenings of the children’s teeth to measure dmfs. Examinations were conducted using a dental mouth mirror and an A-dec LED Dental Light (Model 576L, Newberg, Oregon 97132) attached to the dental chair. The children’s teeth were brushed and dried with gauze prior to visual examination. Dental caries detection and the measurement criteria used in this study were those described by Pitts (34). The dmfs findings were recorded using an electronic dental research record system designated as CARIN (CAries Research Instrument) specifically designed for documentation of the dmfs measure.

### Measures

The BRFQ survey questions related to caregiver knowledge and behavior were included and analyses used baseline BRFQ and oral exam data to assess validity of items designed to measure the five subscales addressed by the EHBM (perceived susceptibility, perceived severity, perceived benefits, perceived barriers, and self-efficacy).

#### Extended Health Belief Model

Seventeen survey items measured the four main constructs from the original HBM encompassing perceived susceptibility, severity, benefits, and barriers (22, 23). Items were adapted from four sources to capture beliefs toward specific behaviors recommended as part of the intervention (35, 36). A Likert-type scale was used for all item responses and ranged from 1 (“Strongly Disagree”) to 5 (“Strongly Agree”). The average of non-missing items associated with each construct was computed, with larger numbers indicating a greater degree of each construct.

#### Self-Efficacy

Ten survey items measured caregivers’ oral health self-efficacy (37, 38). Items were adapted from Reisine’s Dental Confidence Questionnaire (39) or newly developed, and used a Likert-type scale of 1–5, where 1 indicated the caregiver was “not at all sure” she could engage in a given behavior and 5 indicated she was “extremely sure.” For analysis, the average of the self-efficacy items was computed.

#### Oral Health Knowledge

Fourteen questions assessed caregivers’ knowledge of recommended oral health behaviors. Validity of these items was described in an earlier report (40). Responses were coded as correct or incorrect (“don’t know” responses were identified as incorrect). Overall oral health knowledge was measured as the percentage of questions answered correctly based on a range of 0–100%.

#### Oral Health Behavior

Twelve questions, which were previously validated (40), assessed caregivers’ oral health behavior. For each item, responses were coded as adherent or non-adherent with adherent defined as following recommended oral health behavior as defined by the study instrument. A behavioral adherence score was computed representing the percentage of behaviors for which caregivers were adherent.

#### Indicators of Oral Health

Using oral examination data, a score for dmfs was computed for each child at the time of their routine dental visit at the dental clinic. Scoring for dmfs was based on the number of decayed tooth surfaces, missing teeth due to caries, and the number of filled tooth surfaces in any primary tooth. Missing teeth were scored as four surfaces for anterior teeth and five surfaces for posterior teeth. Early childhood caries was defined as a dmfs score >1 in a child under the age of 6 years.

#### Participant Characteristics

Items included age (mother and child), gender (child), maternal employment status and educational attainment, household income, household minors, and years in the household. In analyses, employment was coded as a dichotomous variable indicating whether the participant was employed at least part-time (32 h). Education was coded using a dichotomous scale indicating whether the participant completed high school. Income was measured as the total income of all household members ranging from $10,830 to ≥$37,010.

#### Knowledge on Dental Utilization

Five items measured maternal knowledge on utilization of oral health services for their children.

#### Acculturation

Preference for English or Spanish as the primary language was used as a proxy measure of participant’s level of acculturation. Acculturation was coded using a dichotomous scale with a participant preference for Spanish designated as low acculturation status.

### Data Analysis

For descriptive statistics, categorical variables were summarized as counts and percentages and continuous variables were summarized as means and SDs. Associations between language and each variable were tested using *t*-tests and Fisher’s exact tests. The associations between independent variables and dmfs were modeled using negative binomial regression. Bivariate associations and associations adjusted for age, gender, and language were tested. Data cleaning and analysis were conducted using R version 3.3.3 (41).

Participant’s sociodemographic characteristics were summarized as the mean and SD for continuous variables and count and percent for categorical variables. For variables with missing data, the number of responding participants was included prior to the mean or count. Standardized item-total correlation (ITC) was used to assess internal consistency reliability between each item and its subscale. Values of 0.30 or greater were considered to demonstrate sufficient consistency with the overall subscale. The standardized form of Cronbach’s alpha was used to assess overall internal consistency of each scales, where values of 0.7 or higher reflected good consistency.

Simple linear regression was used to assess the association between sociodemographic characteristics and the HBM and self-efficacy subscales. Multiple linear regression (adjusted for age, gender, and primary language) was used to test for associations between subscales and the convergent measure. Knowledge scores were modeled as predictors of the HBM and self-efficacy subscales, while behavior and dmfs were modeled as outcomes with HBM and self-efficacy as predictors. An interaction term between language and each covariate of interest was separately tested in these models. A significance level of 0.05 was used in all hypothesis tests and all confidence intervals were at the 95% level. All data cleaning and analyses were conducted in R version 3.3.3 (41). ITC and Cronbach’s alpha were calculated using the psych package (42).

## Results

### Demographic Characteristics

A total of 100 Latino mother-–child dyads were enrolled in the study, and survey data and dmfs scores were collected for 99 dyads (Table 1). Mean age of participating children was 4.0 + 1.1 years and 46.5% were female. Mean age of participating mothers was 31.4 + 6.6 years, 60.6% had at least a high school education, and 35.4% were employed. The median household income was $18,310, and mean years in the household was 4.3.

**Table 1:** Demographic characteristics.

	Overall (*N* = 99)
**Child’s age**	
Mean (SD)	99; 3.99 (1.11)
**Child’s gender**	
Male	53 (53.54%)
Female	46 (46.46%)
**Maternal age**	
Mean (SD)	87; 29.54 (9.62)
**Maternal education**	
Less than HS	37/94 (39.36%)
HS or more	57/94 (60.64%)
**Maternal employment**	
Employed	35 (35.35%)
Not employed	64 (64.65%)
**Household income**	
$10,830–$14,569	4/41 (9.76%)
$14,570–$18,309	4/41 (9.76%)
$18,310–$22,049	5/41 (12.20%)
$22,050–$25,789	5/41 (12.20%)
$25,790–$29,529	15/41 (36.59%)
$29,530–$33,269	2/41 (4.88%)
$33,270–$37,009	4/41 (9.76%)
$37,010	2/41 (4.88%)
**Household size**	
Mean (SD)	91; 4.75 (1.42)
**Household minors**	
Mean (SD)	76; 2.66 (1.35)
**Household years**	
Mean (SD)	98; 4.27 (3.51)

### Associations between Demographic Variables and EHBM Subscales

Application of simple linear regression models demonstrated significant differences in the barriers, benefits, and susceptibility subscales (Table 2: continuous variables, Table 3: categorical variables). Specifically, participants with at least a high school education (Table 2) perceived fewer barriers, greater benefits, and greater susceptibility than participants with less education (*P* = 0.004, *P* = 0.048, and *P* = 0.046, respectively). There was also a significant association between years in the household and benefits, with the benefits subscale increasing 0.05 for each additional year in the household (*P* = 0.042).

**Table 2:** Associations between continuous demographic variables and Extended Health Belief Model subscales.

Covariate	Term^a^	Health Belief Model (HBM) barriers	HBM benefits	HBM severity	HBM susceptibility	Self-efficacy
Education	HS or more	1.92 (1.75, 2.08)	4.39 (4.16, 4.62)	4.29 (4.05, 4.52)	3.28 (3.06, 3.51)	4.31 (4.14, 4.47)
	Less than HS	2.31 (2.11, 2.52)	4.02 (3.73, 4.31)	3.94 (3.65, 4.24)	2.91 (2.63, 3.20)	4.06 (3.85, 4.27)
	*P*-value	***P* = 0.004**	***P* = 0.048**	*P* = 0.075	***P* = 0.046**	*P* = 0.072
Employed	No	2.14 (1.97, 2.31)	4.23 (3.99, 4.46)	4.10 (3.88, 4.33)	3.20 (2.97, 3.42)	4.09 (3.92, 4.27)
	Yes	2.05 (1.82, 2.28)	4.15 (3.83, 4.47)	4.23 (3.92, 4.53)	3.01 (2.71, 3.32)	4.30 (4.07, 4.54)
	*P*-value	*P* = 0.533	*P* = 0.714	*P* = 0.507	*P* = 0.335	*P* = 0.159
Gender	Female	1.98 (1.78, 2.18)	4.22 (3.95, 4.50)	4.10 (3.83, 4.36)	3.09 (2.82, 3.35)	4.25 (4.04, 4.46)
	Male	2.21 (2.03, 2.40)	4.18 (3.92, 4.44)	4.19 (3.94, 4.44)	3.17 (2.93, 3.42)	4.10 (3.90, 4.29)
	*P*-value	*P* = 0.096	*P* = 0.810	*P* = 0.621	*P* = 0.638	*P* = 0.301

*From univariate regression models, with HBM subscales as outcomes*.

*All bold font reflects significant values*.

*^a^HS denotes high school*.

**Table 3:** Associations between categorical demographic variables and Extended Health Belief Model subscales.

Covariate^a^	Health Belief Model (HBM) barriers	HBM benefits	HBM severity	HBM susceptibility	Self-efficacy
Age	0.06 (−0.06, 0.19)	−0.04 (−0.21, 0.13)	−0.09 (−0.26, 0.07)	−0.03 (−0.19, 0.14)	−0.03 (−0.16, 0.10)
	*P* = 0.319	*P* = 0.651	*P* = 0.262	*P* = 0.735	*P* = 0.640
HH income	−0.01 (−0.12, 0.11)	−0.04 (−0.14, 0.05)	−0.07 (−0.23, 0.10)	0.02 (−0.13, 0.16)	−0.01 (−0.14, 0.11)
	*P* = 0.869	*P* = 0.352	*P* = 0.408	*P* = 0.803	*P* = 0.820
HH minors	0.02 (−0.09, 0.13)	0.02 (−0.11, 0.15)	−0.04 (−0.20, 0.11)	0.06 (−0.09, 0.21)	0.02 (−0.10, 0.13)
	*P* = 0.689	*P* = 0.772	*P* = 0.567	*P* = 0.452	*P* = 0.750
HH size	0.01 (−0.08, 0.11)	0.00 (−0.11, 0.12)	−0.07 (−0.21, 0.06)	−0.01 (−0.14, 0.12)	−0.05 (−0.14, 0.05)
	*P* = 0.791	*P* = 0.976	*P* = 0.284	*P* = 0.879	*P* = 0.315
Years in HH	0.01 (−0.03, 0.05)	0.05 (0.00, 0.10)	−0.02 (−0.08, 0.03)	0.00 (−0.05, 0.05)	0.01 (−0.03, 0.05)
	*P* = 0.762	***P* = 0.042**	*P* = 0.346	*P* = 0.998	*P* = 0.568
Maternal age	0.02 (−0.00, 0.04)	−0.01 (−0.03, 0.01)	−0.01 (−0.04, 0.02)	−0.01 (−0.04, 0.02)	−0.01 (−0.04, 0.01)
	*P* = 0.098	*P* = 0.440	*P* = 0.552	*P* = 0.459	*P* = 0.198

*From univariate regression models, with HBM subscales as outcomes*.

*All bold font reflects significant values*.

*^a^HH denotes household*.

### Association of EHBM Subscales with Convergent Measures

Application of multiple linear regression models demonstrated that oral health knowledge was significantly associated with increased self-efficacy and increased benefits (Table 4). Additionally, knowledge on dental utilization was associated with these same outcomes as well as susceptibility. All associations were positive, demonstrating increased concern for each subscale with increasing knowledge. No significant associations were found between the EHBM subscales and respondent behavior or dmfs.

**Table 4:** Association of Extended Health Belief Model (EHBM) subscales with convergent measures.

	Self-efficacy	Health Belief Model (HBM) severity	HBM barriers	HBM susceptibility	HBM benefits
Behavior	−0.17 (−4.85, 4.51)	1.67 (−1.87, 5.21)	0.37 (−4.69, 5.43)	−1.46 (−5.10, 2.17)	−2.82 (−6.13, 0.49)
	*P* = 0.944	*P* = 0.352	*P* = 0.886	*P* = 0.425	*P* = 0.094
dmfs	1.80 (−2.96, 6.57)	−2.56 (−6.16, 1.04)	0.49 (−4.68, 5.66)	2.90 (−0.77, 6.58)	2.89 (−0.49, 6.27)
	*P* = 0.454	*P* = 0.161	*P* = 0.853	*P* = 0.120	*P* = 0.093
Oral health knowledge	0.02 (0.00, 0.03)	0.01 (−0.01, 0.03)	−0.01 (−0.02, 0.01)	0.01 (−0.01, 0.03)	0.02 (0.00, 0.04)
	***P* = 0.016**	*P* = 0.170	*P* = 0.433	*P* = 0.415	***P* = 0.021**
Knowledge on dental utilization	0.26 (0.07, 0.45)	0.13 (−0.12, 0.39)	−0.15 (−0.33, 0.02)	0.46 (0.23, 0.69)	0.82 (0.61, 1.04)
	***P* = 0.007**	*P* = 0.310	*P* = 0.088	***P* < 0.001**	***P* < 0.001**

*Presents regression coefficients and (*P* values) from ordinary least squares regression analyses that assessed the relationship of the independent variables (EHBM subscales) with dependent variables (convergent measures). For the oral health knowledge score, knowledge was the independent variable and the EHBM subscales were the dependent variables. All analyses controlled for age, gender, education, and income*.

*All bold font reflects significant values*.

### Demographics, Scales, and dmfs Descriptive Statistics by Primary Language

Sixty-six percent of participating children had caries experience (dmfs > 0) (Table 5). More acculturated maternal participants had higher educational attainment (*P* = 0.0342) and dental utilization knowledge (*P* = 0.0024) compared with less acculturated participants. In relation to the EHBM constructs and expected direction of the model, more acculturated participants had higher scores for perceived susceptibility (*P* = 0.0080) and lower scores for perceived barriers (*P* = 0.0002) and higher scores for self-efficacy (*P* = 0.0043). Contrary to expectations, more acculturated maternal participants had lower scores for perceived benefits (*P* = 0.01951) and borderline scores for perceived severity (*P* = 0.0574).

**Table 5:** Demographics, scales, and dmfs descriptive statistics by primary language.

Variable^a^	Value	English	Spanish	*P*-value
Age (years)		3.94 ± 1.09	4.05 ± 1.15	*P* = 0.6232
dmfs		7.56 ± 12.11	15.20 ± 21.48	***P* = 0.0461**
Child gender	Female	27 (45.8%)	19 (47.5%)	*P* = 1.0000
	Male	32 (54.2%)	21 (52.5%)	
Maternal age		55; 28.20 ± 9.77	32; 31.84 ± 9.05	*P* = 0.0830
Mothers education	HS or more	39 (66.1%)	18 (45.0%)	***P* = 0.0342**
	Less than HS	17 (28.8%)	20 (50.0%)	
	(Missing)	3 (5.1%)	2 (5.0%)	
Household size		56; 4.66 ± 1.53	35; 4.89 ± 1.23	*P* = 0.4426
				
Household minors		44; 2.61 ± 1.42	32; 2.72 ± 1.28	*P* = 0.7362
Years in household		4.58 ± 3.67	39; 3.79 ± 3.25	*P* = 0.2716
Oral health behavior		47.13 ± 14.98	41.76 ± 16.18	*P* = 0.0988
Oral health knowledge		87.51 ± 7.65	85.67 ± 12.18	*P* = 0.3995
Knowledge on dental utilization		3.67 ± 0.51	3.15 ± 0.93	***P* = 0.0024**
**Extended Health Belief Model**				
Self-efficacy		4.34 ± 0.59	3.91 ± 0.79	***P* = 0.0043**
Perceived severity		4.29 ± 0.94	3.94 ± 0.82	*P* = 0.0574
Perceived barriers		1.89 ± 0.61	2.42 ± 0.68	***P* = 0.0002**
Perceived susceptibility		3.34 ± 0.75	2.83 ± 1.03	***P* = 0.0080**
Perceived benefits		4.31 ± 0.67	4.03 ± 1.24	*P* = 0.1951

*All bold font reflects significant values*. ^a^Continuous variables are presented as mean ± SD. Categorical variables are presented as “count (percent).”

### EHBM Subscale and Item Summary and ITC

The correlation of each item with its subscale (ITC) was considered acceptable if 0.3 or higher (Table 6). All EHBM items were acceptable except for two items (HBM3, HBM6) in the barriers subscale (ITC = 0.25, 0.39, respectively) suggesting these items were inconsistent with other items in the subscale. All other individual items were sufficiently correlated with total scores to suggest they are consistent with each subscales’ concept. The benefits subscale and the self-efficacy scale showed good consistency with Cronbach’s alpha values being greater than 0.7 (0.87 and 0.82). The barriers, severity and susceptibility were less than 0.7 (0.07, 0.12, 0.31). Average responses for the benefits, severity, and self-efficacy subscales were 4.20, 4.15, and 4.17 indicating areas of concern for participants. Barriers and susceptibility were less of a concern with average scores of 2.11 and 3.13, respectively.

**Table 6:** Extended Health Belief Model subscale and item summary and item-total correlation (ITC).

Scale	Item	Item label	*N*; mean (SD)	ITC
Barriers	HBM2	It would be hard to take my child for regular dental checkups	93; 1.77 (1.36)	0.70
	HBM3	It is hard to keep my child from eating sweet foods and drink	97; 2.23 (1.23)	0.25
	HBM6	I have no trouble making sure that my child’s teeth are brushed the last thing before bed	96; 1.92 (1.51)	0.39
	HBM9	It’s inconvenient to have fluoride varnish put on my child’s teeth	87; 2.44 (1.65)	0.46
	HBM11	It’s easy to make sure that my child’s teeth are brushed with fluoride toothpaste twice a day	95; 2.08 (1.36)	0.50
Benefits	HBM18	My child is unlikely to get cavities if his/her teeth are brushed with fluoride toothpaste twice a day	87; 4.07 (1.31)	0.75
	HBM19	My child is unlikely to get cavities if he/she goes to the dentist for regular checkups	97; 3.88 (1.36)	0.77
	HBM20	My child is unlikely to get cavities if I keep him/her from eating a lot of sugary food and drinks	95; 4.42 (1.06)	0.78
	HBM21	My child is unlikely to get cavities if an adult helps brush his/her teeth until at age 6	96; 4.43 (1.03)	0.90
	HBM22	My child is unlikely to get cavities if a dentist or other care provider puts fluoride varnish on his/her teeth	88; 4.25 (1.16)	0.85
Severity	HBM1	Dental problems could be serious for a child	97; 4.43 (1.33)	0.44
	HBM5	Having bad teeth does not affect a child’s everyday life	97; 3.79 (1.66)	0.66
	HBM8	Dental problems are not as important as other health problems	97; 4.29 (1.30)	0.71
Susceptibility	HBM4	Most children get cavities	92; 3.55 (1.36)	0.62
	HBM7	My child will probably get cavities in next few years	91; 2.08 (1.00)	0.56
	HBM10	Children can get cavities as soon as there first tooth comes in	90; 3.78 (1.52)	0.59
	HBM12	It is not likely that my child will have problems with his/her teeth	88; 3.07 (1.32)	0.51
Self-efficacy	SE1	Carefully check your child’s teeth and gums every month for spots and problems?	93; 3.94 (1.17)	0.53
How sure are you that you can …	SE2	Take your child to the dentist for regular checkups?	99; 4.78 (0.71)	0.64
	SE3	Always use fluoride toothpaste when brushing your child’s teeth?	93; 4.41 (1.01)	0.61
	SE4	Make sure that your child does not eat or drink anything other than water after the teeth and gums are cleaned at bedtime?	97; 4.26 (1.13)	0.64
	SE5	Keep your child from eating frequent sweets? (cake/candy)	97; 3.86 (1.19)	0.63
	SE6	Keep your child from putting anything in his/her mouth that has been in someone else’s mouth?	98; 3.84 (1.30)	0.75
	SE7	Have fluoride varnish put on your child’s teeth by a dentist or other health care provider?	94; 4.07 (1.10)	0.47
	SE8	Keep your child from drinking sugary drinks like soda, pop or Kool-Aid?	98; 3.67 (1.26)	0.67
	SE9	Avoid putting your child to bed with a bottle or sippy cup with anything other than water in it?	97; 4.60 (1.01)	0.61
	SE10	Make sure your child’s teeth are brushed twice a day?	98; 4.38 (1.01)	0.67
Scale	Cronbach’s alpha	Mean (SD)		
HBM barriers	0.07	2.11 (0.69)		
HBM benefits	0.87			
HBM severity	0.12			
HBM susceptibility	0.31	3.13 (0.90)		
HBM self-efficacy	0.82	4.17 (0.71)		

*ITC of 0.30 or higher was considered to reflect an acceptable degree of association between an item and the total score for its subscale. Cronbach’s alpha of 0.70 or higher reflected an acceptable degree of consistency among items in a subscale*.

### Associations between Oral Health Knowledge and EHBM Subscales for Each Primary Language

Multiple regression models were extended to include the interaction of language and each primary predictor to determine whether the primary language affects associations between each of the knowledge, behavior, dmfs measures, and EHBM subscales (Table 7). Due to the large number of models, only significant results are included. Two of the models showed significant interaction effects for the primary language. The main effects model (without the interaction of language) of knowledge and EHBM barriers showed no significant association. The addition of the interaction of language resulted in a significant difference for Spanish-speaking participants in the association between knowledge and barriers, thereby suggesting that language acts as an effect modifier. Children of Spanish-speaking participants have a statistically significant negative (*P* = 0.038) association, while English-speaking participants have a non-significant but positive association (*P* = 0.152). A significant interaction was also found in the association between utilization knowledge and the benefits subscale in Spanish-speaking participants but not in English-speaking participants. For every 1 U increase in dental utilization knowledge for Spanish-speaking participants, an increase of 0.72 U was found for the benefits subscale compared to English-speaking participants. In Spanish-speaking participants, this was equivalent to an increase of 1.04 for the benefits Likert subscale for each unit increase in knowledge utilization (*P* < 0.0001).

**Table 7:** Associations between oral health knowledge and Extended Health Belief Model subscales for each primary language.

Outcome	Covariate	Comparison	Estimate	Lower	Upper	*P*-value
Health Belief Model (HBM)—barriers	Knowledge	English slope	0.02	−0.01	0.04	*P* = 0.152
		Spanish slope	−0.02	−0.03	−0.00	***P* = 0.038**
HBM—benefits	Utilization knowledge	English slope	0.32	−0.05	0.70	*P* = 0.088
		Spanish slope	1.04	0.80	1.29	***P* < 0.001**

*All bold font reflects significant values*.

## Discussion

**Figure 1:**
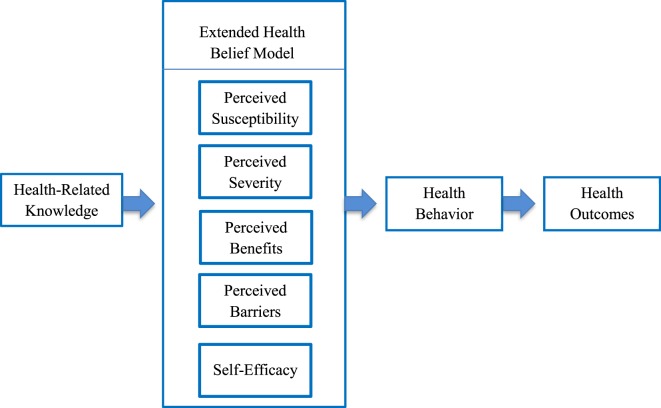
Extended Health Belief Model.

Latino children experience one of the highest rates of early childhood caries (3), as reflected in this study with the prevalence approaching 70%. Application of the EHBM theoretical framework in relation to individual and cultural maternal factors offered insight for existing oral health disparities in young children. Per the proposed direction of the model, maternal knowledge was expected to be a predictor of HBM subscales and self-efficacy, while the HBM subscales and self-efficacy were expected to be predictors of maternal oral health behaviors and children’s’ oral health outcomes or dmfs (Figure 1). As anticipated, mothers with increased knowledge (including dental utilization) perceived that there were greater benefits from adherence with recommended oral health behavior and had greater confidence in ability to manage their children’s oral health. Additionally, mothers with increased dental utilization knowledge perceived their children as more susceptible to developing early childhood caries. Contrary to expectations, Latino mothers with increased knowledge did not perceive early childhood caries as a serious condition or that children were susceptible to developing cavities and reported higher barriers. Findings were similar to other studies involving Latina mothers, in which higher knowledge did not translate to greater adherence with recommended oral health behaviors or improved oral health outcomes among children (43). Based on findings and the directional basis of the EHBM, strategies focused on maternal knowledge and behavior rather than knowledge alone may have greater potential to improve oral health outcomes.

In relation to the EHBM constructs, education was the strongest predictor of maternal oral health beliefs. Educational attainment was associated with all constructs of the EHBM excluding perceived severity. Mothers with higher educational attainment viewed their children as more susceptible to cavities, reported greater benefits to and fewer barriers to recommended oral health behavior, and were more confident in their ability to engage in optimal oral health behavior. Latina mothers with higher educational status, however, did not perceive cavities as more serious. Variation in the severity subscale may be related to cultural influences and practices in Latino mothers. In previous qualitative studies, Latina mothers reported they did not perceive that dental decay was a condition that affected young children (43, 44). Findings highlight the importance of identifying and addressing specific cultural beliefs that are counter to optimal oral health in young children.

Study outcomes also reflected that the duration in the household significantly influenced maternal oral health beliefs. Longer durations in the household positively influenced maternal perceptions regarding greater benefits to adherence with recommended oral health behaviors. Other studies suggest that frequency of residential moves including immigrations affect children’s oral health outcomes (4). Dislocations in residence decrease stability within families and disrupt access to health-care services and benefits. Specific guidance on maintaining a dental home for children despite changes in household duration is recommended. Outcomes reinforce the importance of integrated care systems that facilitate access to care and services for disadvantaged children and families.

The inter-relationship between the individual and cultural level factors for participating mothers demonstrated that less acculturated mothers (Spanish-speaking) overall perceived greater barriers to adherence, but perceived fewer barriers as their knowledge increased. In addition, as dental utilization knowledge improved for Spanish-speaking mothers, they perceived greater benefits to adherent oral health behavior compared to English-speaking mothers. Significant findings were not found for maternal behavior and early childhood caries in relation to the EHBM constructs and acculturation status or their inter-relationship. Negative health outcomes may be explained by the concept of inverse care law. The premise of inverse care law implies that individuals and groups with lower health needs experience greater benefits from care compared to those with a higher health needs (45, 46). Other contributory factors include a higher prevalence of comorbidities involving psychological distress, health literacy, and fatalism. Inability to manage multiple comorbidities may lead to a more selective focus that may not include dental caries in children (47). Low caregiver health literacy has been associated with reduced ability to accomplish child-related tasks (48). Enhancing caregivers’ health literacy and other beliefs may improve use of health-care systems and oral health outcomes for children (30, 44).

In summary, the results from this study suggested that the items assessing the EHBM theoretical constructs are valid as measures of maternal factors influencing children’s oral health outcomes in a Latino population. Study limitations included a smaller sample from a clinically based population in a single location that may not reflect all Latino communities. The cultural and language orientation of participants may have influenced responses to the translated items due to subtle differences in interpretation. Familiarity with the community, use of language consultants, and pilot studies may enable more accurate responses with translated instruments (30). Additional studies are warranted to determine whether these measures fit expectations regarding the relationship of these theoretical constructs over time. Testing of these measures among a range of Latino groups as well as other socially disadvantaged groups will lend additional support to these measures.

## Author Contributions

All authors (AW, MM, and TT) have made substantial contributions to the design of the work, analysis of the data, and revision of the work and provided final approval. All authors agreed to be accountable for all aspects of the work to ensure questions related to the accuracy or integrity of any part of the work are appropriately investigated and resolved.

## Conflict of Interest Statement

The authors declare that the research was conducted in the absence of any commercial or financial relationships that could be construed as a potential conflict of interest.
